# Supplementary material for: Categorizing prediction modes within low-pLDDT regions of AlphaFold2 structures
Source: bioRxiv. 2025 Jun 7:2025.06.06.658382. Preprint. [Version 1] doi: 10.1101/2025.06.06.658382 (PMC12157579; doi:10.1101/2025.06.06.658382)
Supplement: Supplement 2 [file NIHPP2025.06.06.658382v1-supplement-2.pdf]

bioRxiv preprint doi: https://doi.org/10.1101/2025.06.06.658382; this version posted June 7, 2025. The copyright holder for this preprint (which was not certified by peer review) is the author/funder, who has granted bioRxiv a license to display the preprint in perpetuity. It is made available under aCC-BY 4.0 International license.

Supporting information

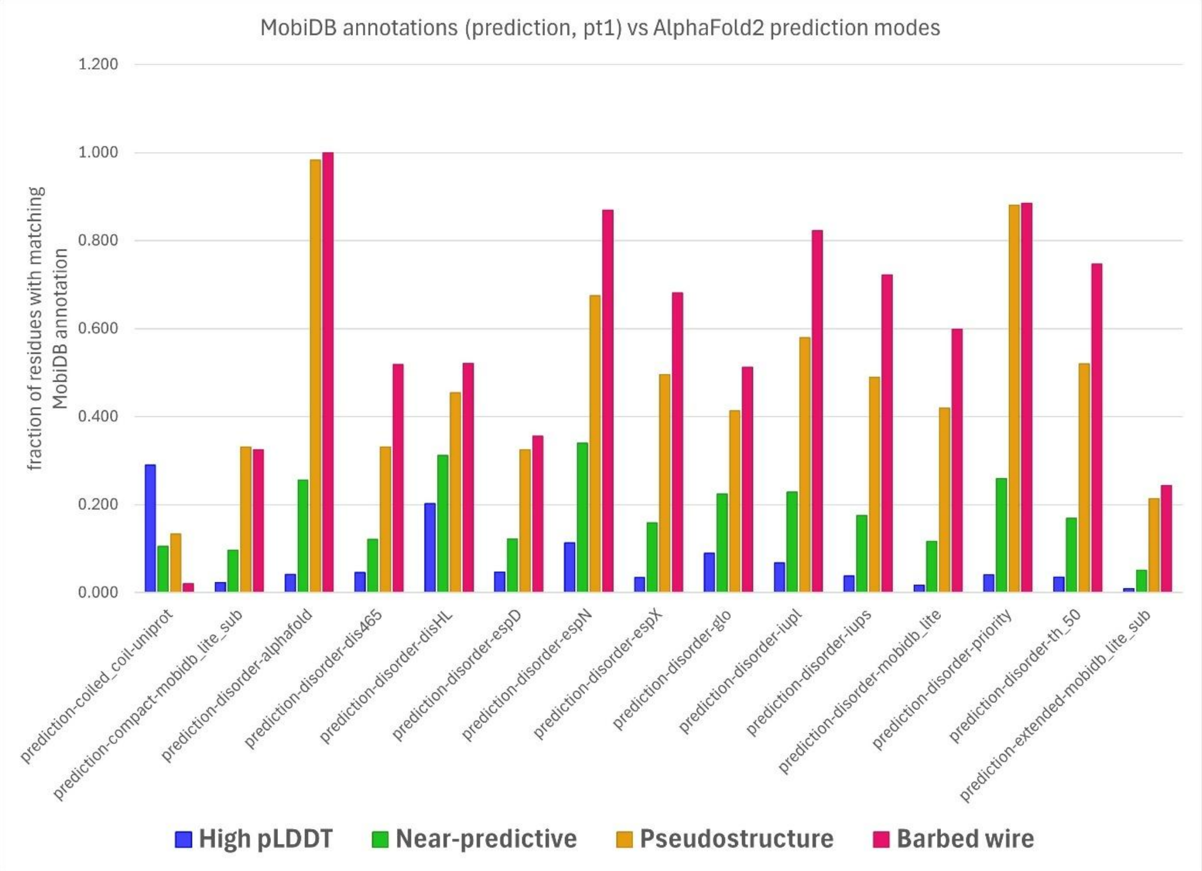

Figure S1 Prediction annotations from MobiDB and their relationships with AlphaFold2 prediction modes. Bar height is the fraction of residues from that prediction mode that were marked with the matching MobiDB annotation. Not all sequences are treated with all annotations, and only residues from duly annotated sequences were considered for each annotation.

These prediction annotations show the general stair-step pattern discussed in the main text. Prediction-disorder-iupl is included in Figure 7 as a representative of this behavior. Correlations with prediction-disorder-alphafold are not considered significant, since that annotation is also interpreting AlphaFold results, rather than directly interpreting the underlying sequence.

bioRxiv preprint doi: https://doi.org/10.1101/2025.06.06.658382; this version posted June 7, 2025. The copyright holder for this preprint (which was not certified by peer review) is the author/funder, who has granted bioRxiv a license to display the preprint in perpetuity. It is made available under aCC-BY 4.0 International license.

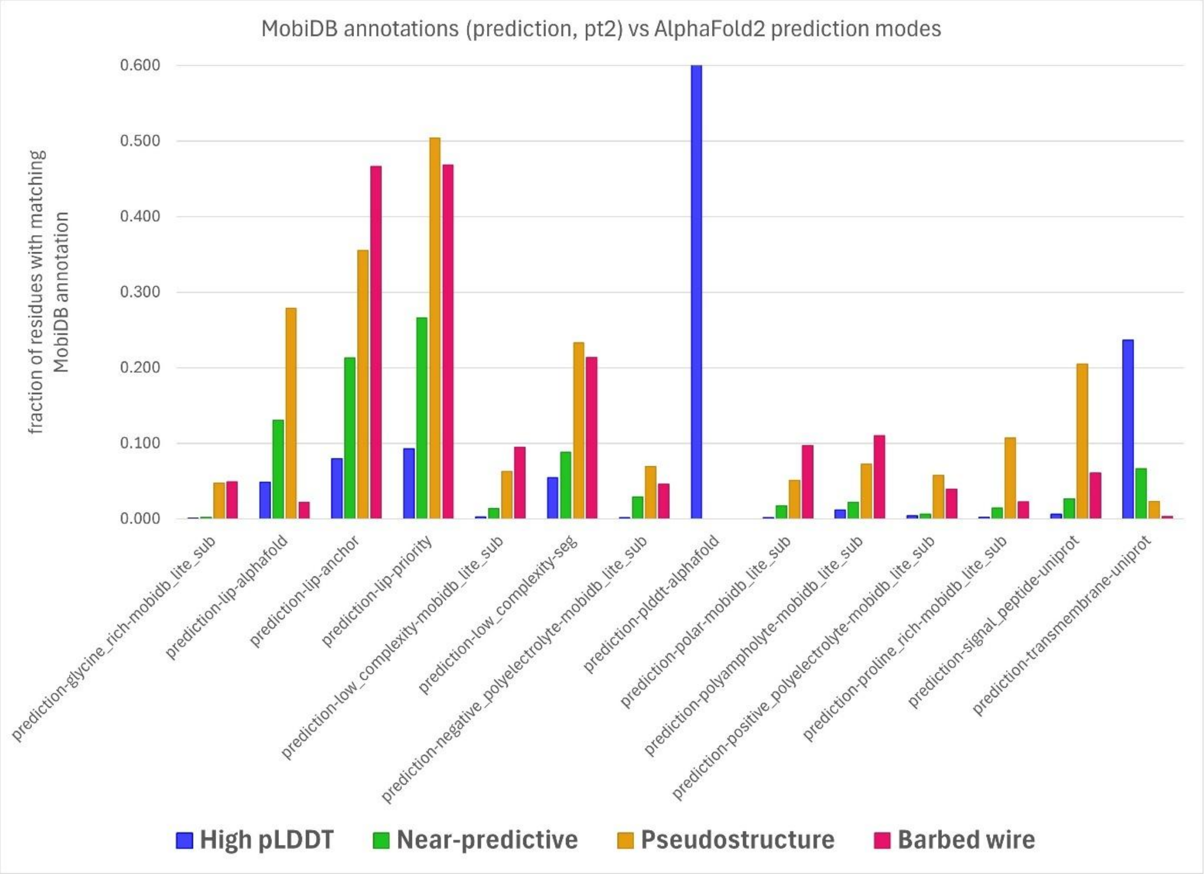

Figure S2 Additional prediction annotations from MobiDB and their relationships with AlphaFold2 prediction modes. Bar height is the fraction of residues from that prediction mode that were marked with the matching MobiDB annotation. Not all sequences are treated with all annotations, and only residues from duly annotated sequences were considered for each annotation.

The y-axis is truncated at 0.6, otherwise the trivial prediction-plddt-alphafold result (which goes to 1.0) would dominate. Prediction-low_complexity-seg, prediction-proline_rich-mobidb_lite_sub, and prediction-signal_peptide-uniprot are included in Figure 7. The preference of prediction-transmembrane-uniprot for high-pLDDT residues partly reflects the tendency of membrane insertion helices to be predicted in the *unpacked high-pLDDT* mode, which is included in the high-pLDDT category for these plots.

bioRxiv preprint doi: https://doi.org/10.1101/2025.06.06.658382; this version posted June 7, 2025. The copyright holder for this preprint (which was not certified by peer review) is the author/funder, who has granted bioRxiv a license to display the preprint in perpetuity. It is made available under aCC-BY 4.0 International license.

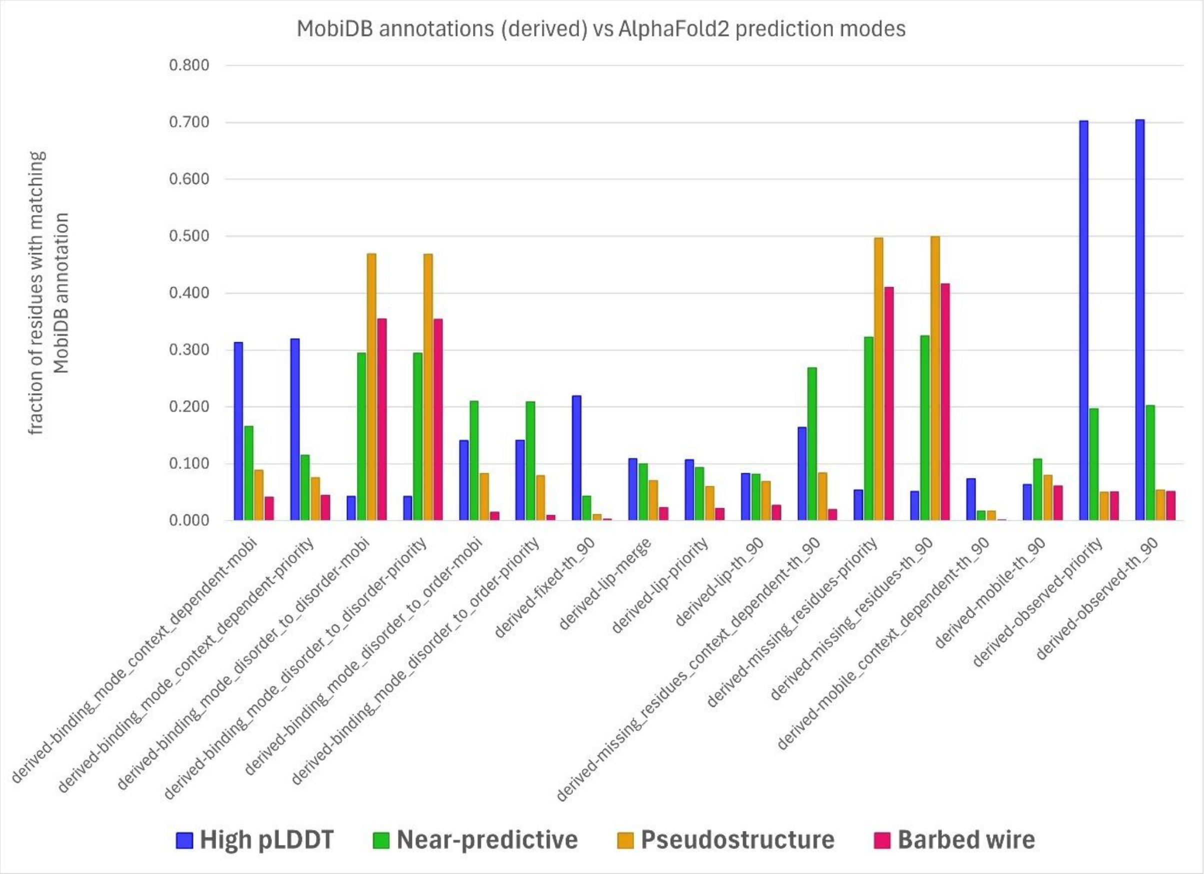

Figure S3 Derived annotations from MobiDB and their relationships with AlphaFold2 prediction modes. Bar height is the fraction of residues from that prediction mode that were marked with the matching MobiDB annotation. Not all sequences are treated with all annotations, and only residues from duly annotated sequences were considered for each annotation.

Derived-binding_mode_disorder_to_disorder-mobi and derived-binding_mode_disorder_to_order-mobi are included in Figure 7. Derived-missing_residues-priority is related to residues omitted from experimentally-solved structures. That *near-predictive residues* are frequently missing from their solved structures confirms our difficulty in finding experimentally-solved versions of *near-predictive* regions to check prediction accuracy.

bioRxiv preprint doi: https://doi.org/10.1101/2025.06.06.658382; this version posted June 7, 2025. The copyright holder for this preprint (which was not certified by peer review) is the author/funder, who has granted bioRxiv a license to display the preprint in perpetuity. It is made available under aCC-BY 4.0 International license.

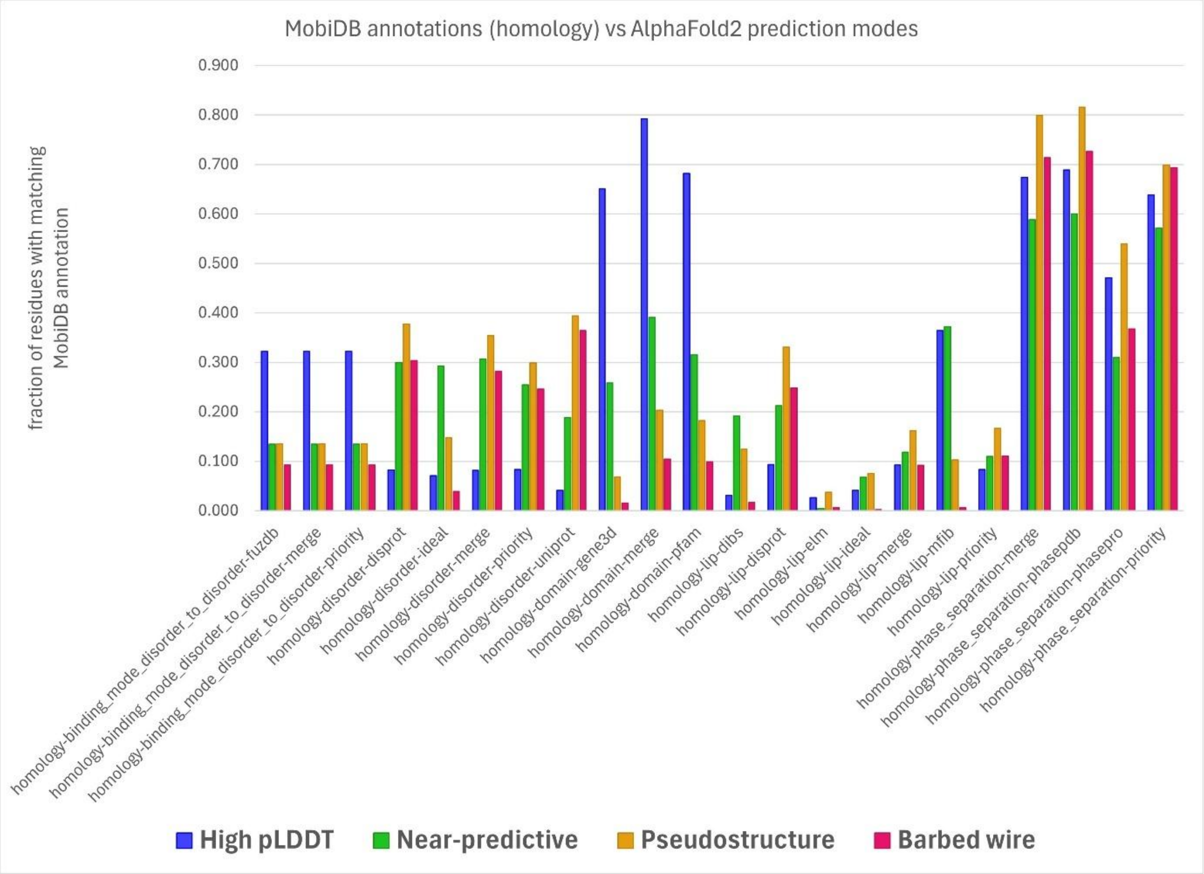

Figure S4 Homology annotations from MobiDB and their relationships with AlphaFold2 prediction modes. Bar height is the fraction of residues from that prediction mode that were marked with the matching MobiDB annotation. Not all sequences are treated with all annotations, and only residues from duly annotated sequences were considered for each annotation.

IDEAL is an annotation associated with conditional order; homology-disorder-ideal shows a strong association with *near-predictive*, a lesser association with *pseudostructure*, and very little association with *barbed wire*. This pattern is similar to derived-binding_mode_disorder_to_order-mobi in Figure S3 and supports our conjecture that the *near-predictive* mode is how AlphaFold2 predicts many conditionally ordered IDRs.

bioRxiv preprint doi: https://doi.org/10.1101/2025.06.06.658382; this version posted June 7, 2025. The copyright holder for this preprint (which was not certified by peer review) is the author/funder, who has granted bioRxiv a license to display the preprint in perpetuity. It is made available under aCC-BY 4.0 International license.

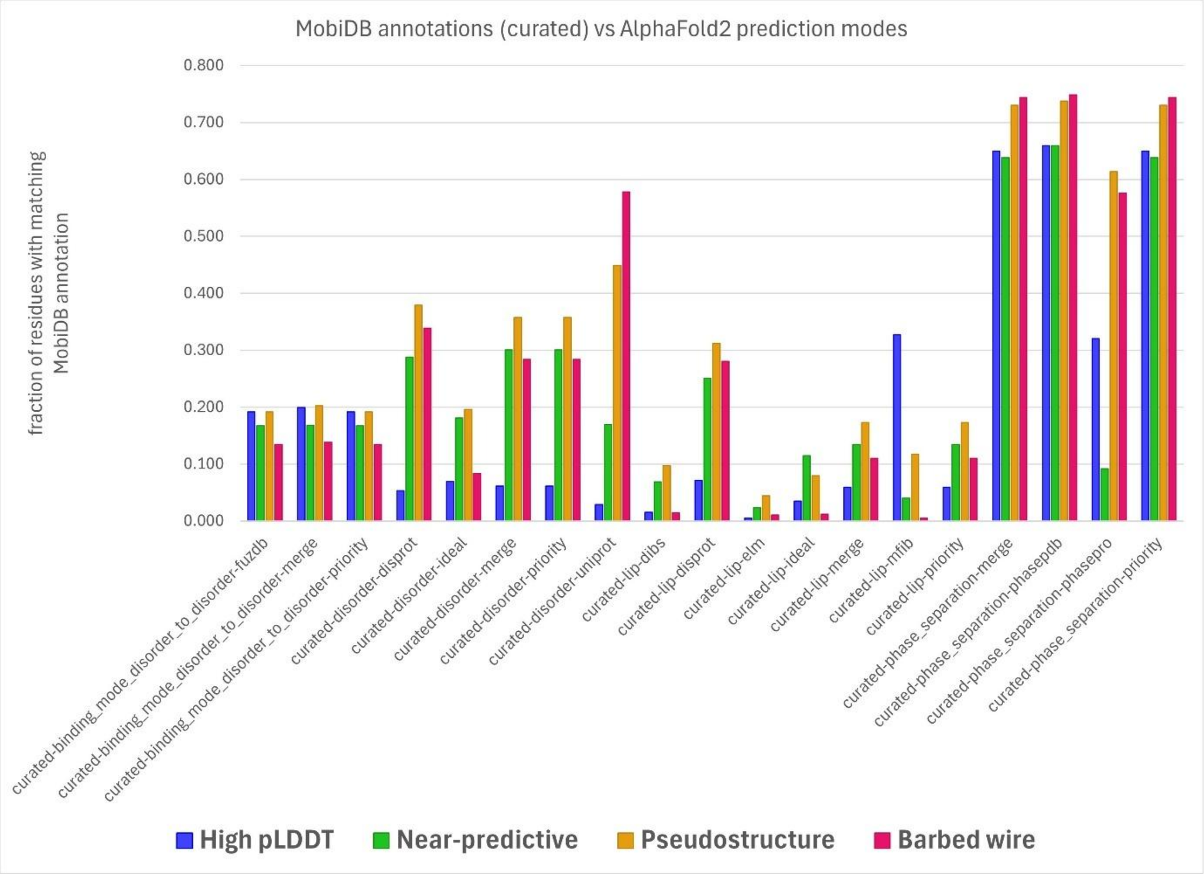

Figure S5 Curated annotations from MobiDB and their relationships with AlphaFold2 prediction modes. Bar height is the fraction of residues from that prediction mode that were marked with the matching MobiDB annotation. Not all sequences are treated with all annotations, and only residues from duly annotated sequences were considered for each annotation.

IDEAL is an annotation associated with conditional order. Curated-disorder-ideal shows a more balanced association of *near-predictive* and *pseudostructure* with conditional order than homology-disorder-ideal in Figure S4 above. This may indicate additional complexity in how conditionally folded IDRs manifest in AlphaFold2 predictions, or it may reflect difficulties in IDEAL's literature-based disorder annotation similar to our own challenges in finding experimentally solved *near-predictive* regions.
